# Thulium laser-assisted laparoscopic management of intrahepatic stones in recurrent pyogenic cholangitis: A case report and review of minimally invasive approaches

**DOI:** 10.1016/j.ijscr.2025.111005

**Published:** 2025-02-02

**Authors:** Hariruk Yodying

**Affiliations:** Department of Surgery, HRH Princess MahaChakri Sirindhorn Medical Center, Faculty of Medicine, Srinakharinwirot University, Nakhon Nayok, Thailand

**Keywords:** Recurrent pyogenic cholangitis, Hepatolithiasis, Laparoscopic surgery, Thulium laser lithotripsy, Intrahepatic stones

## Abstract

**Introduction:**

Recurrent pyogenic cholangitis (RPC) presents significant management challenges, particularly when complicated by large intrahepatic stones. While thulium laser technology has demonstrated excellent results in urological stone treatment, its application in biliary stones remains relatively unexplored. We present a novel approach utilizing laparoscopic choledochoscopy with thulium laser lithotripsy for managing intrahepatic stones in RPC.

**Case presentation:**

A 65-year-old female presented with a two-year history of recurrent right upper quadrant pain, fever, and jaundice. Diagnostic imaging revealed multiple large intrahepatic stones without any liver atrophy or mass lesions. After initial ERCP for acute cholangitis management, the patient underwent laparoscopic choledochoscopy with thulium laser lithotripsy. The procedure included stone fragmentation, extraction, and choledochoduodenostomy for long-term biliary drainage. The procedure was successfully completed with minimal blood loss and no intraoperative complications. At 6-month follow-up, the patient remained asymptomatic with normal liver function, despite small retained stones.

**Discussion:**

This approach combines the benefits of minimally invasive surgery with advanced laser technology. Thulium laser offers potential advantages over conventional lithotripsy methods, including enhanced precision and reduced risk of bile duct injury. The successful outcome in this case suggests that this technique may be a viable option for complex hepatolithiasis in RPC.

**Conclusion:**

Laparoscopic choledochoscopy with thulium laser lithotripsy represents a promising minimally invasive option for managing intrahepatic stones in RPC. While our case demonstrates technical feasibility, long-term follow-up and larger studies are needed to fully evaluate its efficacy.

## Highlights

• First reported case of thulium laser lithotripsy for intrahepatic stones in recurrent pyogenic cholangitis
• Novel combination of laparoscopic choledochoscopy and thulium laser technology demonstrates successful stone fragmentation.
• Minimally invasive approach achieved with minimal blood loss and no intraoperative complications
• Technique offers potential advantages over conventional methods, including enhanced precision and reduced tissue damage

## 1 Introduction

Recurrent pyogenic cholangitis (RPC) is a challenging syndrome characterized by repeated episodes of biliary infection, inflammation, and stone formation, predominantly affecting East and Southeast Asian populations [1]. This condition is often associated with hepatolithiasis, with reported prevalence rates up to 30 % in endemic areas [2]. The pathophysiology involves a complex interplay of biliary infection, cholestasis, and anatomical variations, creating a self-perpetuating cycle of stone formation and biliary damage [3,4].

Long-standing RPC with hepatolithiasis carries significant risks, including progression to secondary biliary cirrhosis and development of cholangiocarcinoma, with reported incidence rates of up to 21.2 % in affected patients [5]. These potential complications underscore the importance of effective management strategies. Moreover, RPC has been identified as an independent poor prognostic indicator for resectable intrahepatic cholangiocarcinoma [6], further emphasizing the need for optimal management.

Traditional approaches for stone clearance of RPC with hepatolithiasis management have included open surgery, percutaneous transhepatic cholangioscopic lithotomy (PTCSL), and various endoscopic techniques [[7], [8], [9]]. However, these methods often have limitations in terms of invasiveness, stone clearance rates, or ability to address concomitant biliary strictures. Open surgical techniques, while effective, are associated with significant morbidity and longer recovery times [10,11]. PTCSL, though less invasive, often requires multiple sessions and has high recurrence rates [12]. Endoscopic approaches like ERCP face challenges in accessing peripheral intrahepatic stones and managing severe biliary strictures [13].

Recent advancements in minimally invasive surgery and laser technology have opened new avenues for RPC treatment. Laparoscopic approaches offer the benefits of reduced postoperative pain and faster recovery [7,14], while laser lithotripsy provides precise stone fragmentation with minimal risk of bile duct injury [15,16]. The emerging Thulium fiber laser (TFL) technology offers potential advantages over the traditionally used Holmium:YAG lasers in terms of ablation efficiency and safety [17].

This case report presents a novel application of laparoscopic choledochoscopy with thulium laser lithotripsy for the management of intrahepatic stones in a patient with RPC. We describe the surgical technique, immediate outcomes, and short-term follow-up, accompanied by a review of current literature on RPC management strategies. Our objective is to evaluate the feasibility and potential benefits of this innovative approach in the context of existing treatment modalities for RPC. This case report has been reported in line with the SCARE 2023 criteria [18].

## 2 Case presentation

A 65-year-old female presented to our emergency department with severe right upper quadrant pain, fever (38 °C), and jaundice. She reported a 2-year history of recurrent episodes of abdominal pain, each lasting several days and occurring approximately every 3–4 months. The patient had no significant medical history apart from these episodes and denied any history of alcohol abuse or hepatitis. There was no history of previous biliary interventions or surgeries.

On physical examination, the patient appeared acutely ill with icteric sclera and marked right upper quadrant tenderness. Vital signs showed tachycardia (110 bpm) and fever. Laboratory tests revealed leukocytosis (WBC 15,000/μL), elevated liver enzymes (AST 150 U/L, ALT 180 U/L), and hyperbilirubinemia (total bilirubin 5.5 mg/dL, direct bilirubin 3.8 mg/dL).

**Fig. 1:**
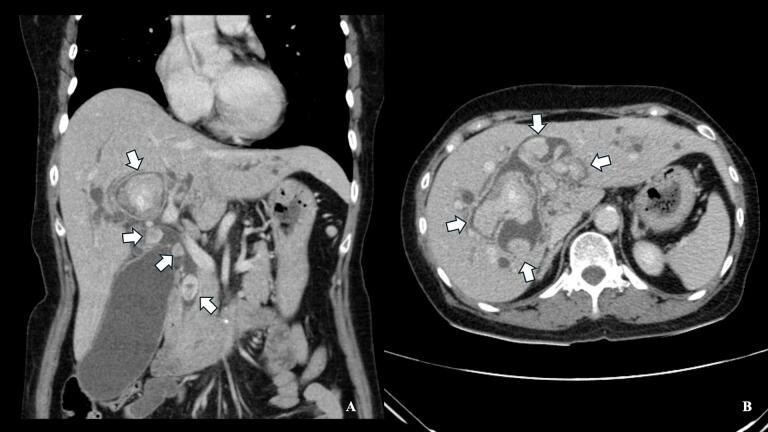
Computed tomography findings in recurrent pyogenic cholangitis. (A) Coronal and (B) axial contrast-enhanced CT images of a 65-year-old female with recurrent pyogenic cholangitis. Multiple hyperdense structures (white arrows) are visible within diffusely dilated intrahepatic ducts and common bile duct, consistent with pigmented stones. Note the widespread biliary dilatation, a hallmark of recurrent pyogenic cholangitis.

**Fig. 2:**
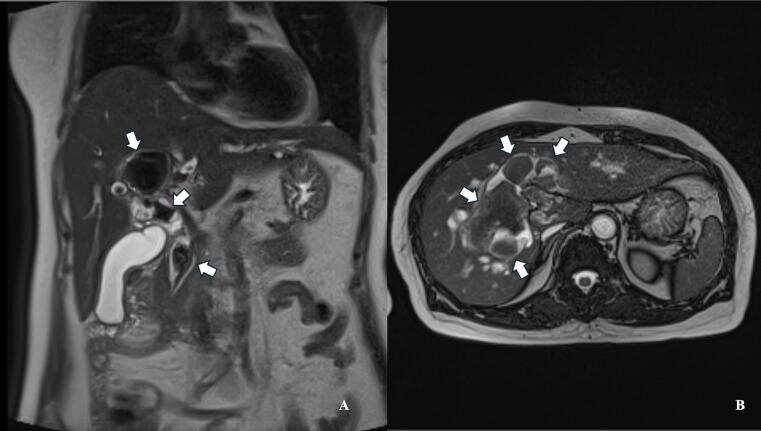
Magnetic resonance imaging of hepatolithiasis. (A) Coronal and (B) axial T2-weighted MRI images demonstrating multiple hypointense filling defects (white arrows) within dilated intrahepatic ducts. The largest stone, measuring 3.3 × 5.6 cm, is visible in the right intrahepatic duct. T2-weighted images provide excellent contrast between the hyperintense bile and hypointense stones.

Abdominal ultrasonography demonstrated dilated intrahepatic bile ducts with hyperechoic foci suggesting calculi. Subsequent contrast-enhanced computed tomography (CT) confirmed the presence of multiple pigmented stones within the diffuse dilated intrahepatic ducts (IHDs) and common bile duct (CBD) without any liver atrophy or mass lesions (Fig. 1). Magnetic resonance imaging (MRI) revealed multiple pigmented stones within the diffuse dilated IHDs and CBD, with the largest stone measuring 3.3 × 5.6 cm in the right IHD (Fig. 2). These imaging findings, combined with the patient's clinical presentation, were consistent with the typical features of RPC as described in current literature [1,2].

The patient initially underwent endoscopic retrograde cholangiopancreatography (ERCP) with stent placement for acute cholangitis management. After resolution of the acute episode, as evidenced by normalization of inflammatory markers and improvement in liver function tests, she was scheduled for elective laparoscopic-assisted surgery. The decision to proceed with laparoscopic choledochoscopy and thulium laser lithotripsy was based on the large size of the intrahepatic stones, the recurrent nature of the disease, and the potential benefits of a minimally invasive approach in terms of recovery [7,19].

## 3 Surgical technique

The procedure was performed under general anesthesia. Four laparoscopic ports were placed: a 12 mm umbilical port for the camera, a 5 mm epigastric port, and two 5 mm right upper quadrant ports. This configuration provided optimal triangulation and unimpeded access to the hepatobiliary system, following established approaches for laparoscopic biliary surgery [10,11].

## 4 Operative approach and initial exploration

**Fig. 3:**
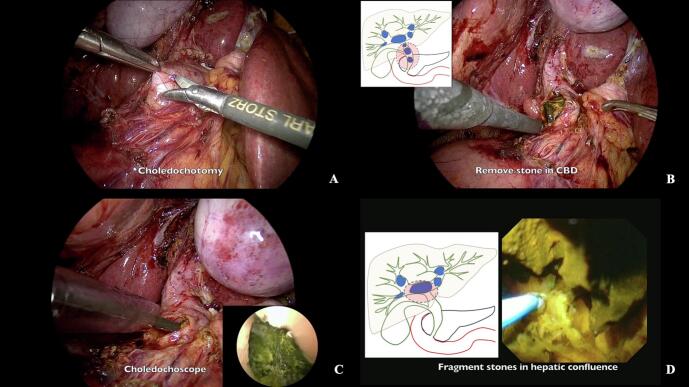
Key steps in laparoscopic choledochoscopy and stone extraction. (A) Laparoscopic view of choledochotomy using scissors. (B) Extraction of small stones from the common bile duct using grasping forceps. (C) Choledochoscopic view of a large intrahepatic stone. (D) Thulium laser fiber (blue light) directed at the stone surface during lithotripsy at the hepatic confluence. Note the precision of the laser application. (For interpretation of the references to colour in this figure legend, the reader is referred to the web version of this article.)

Initial laparoscopic ultrasonography confirmed the location and distribution of intrahepatic stones, providing real-time guidance for surgical planning. A longitudinal choledochotomy was carefully performed on the dilated common bile duct using laparoscopic scissors (Fig. 3A). The previously placed ERCP stent was identified and removed. Initial stone clearance focused on the CBD, where smaller calculi were extracted directly using a combination of grasping forceps and stone baskets (Fig. 3B).

## 5 Choledochoscopy and laser lithotripsy

A flexible choledochoscope was introduced through the choledochotomy, allowing systematic exploration of the biliary tree. Direct visualization revealed multiple large intrahepatic stones, with the largest measuring 3.3 × 5.6 cm in the right hepatic duct (Fig. 3C). For stone fragmentation, we employed a thulium fiber laser system (Olympus SOLTIVE™ SuperPulsed Laser) utilizing two distinct parameter sets optimized for different phases of stone clearance, based on established protocols for thulium laser lithotripsy [15,20,21].

The dusting technique utilized pulse energy of 0.2 J, frequency of 100 Hz, power of 20 W, and pulse width of 1, while larger stone fragmentation employed higher pulse energy of 0.6–1.0 J with reduced frequency of 30 Hz, power of 18 W, and pulse width of 1. This dual-parameter approach allowed for efficient stone breakdown while minimizing the risk of ductal injury through precise energy delivery [16,17].

**Fig. 4:**
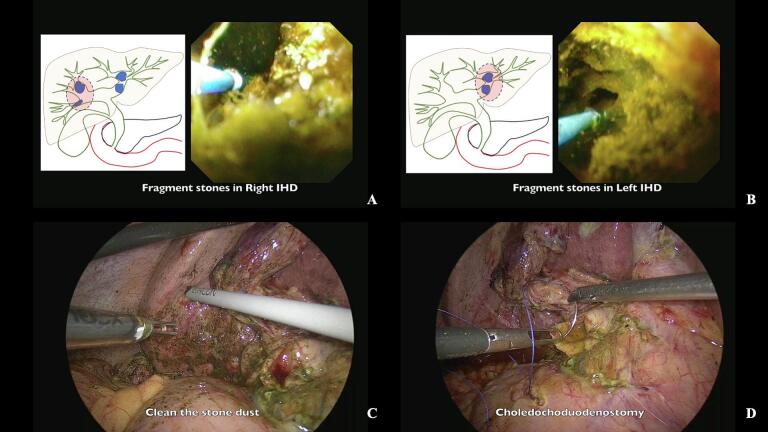
Thulium laser lithotripsy and choledochoduodenostomy cholangioscopic views of stone fragmentation in the (A) right and (B) left intrahepatic ducts using thulium laser. Note the effectiveness of the laser in breaking down large stones. (C) Laparoscopic view of stone dust removal from the subhepatic area using irrigation and suction. (D) Side-to-side choledochoduodenostomy using 3-0 Vicryl absorbable suture, providing long-term biliary drainage.

The laser fiber was applied directly to the stone surface under continuous choledochoscopic visualization (Fig. 3D). Continuous irrigation was maintained throughout the procedure to optimize visualization and facilitate fragment removal. Stone fragments were systematically extracted using basket retrieval devices, with careful attention to preventing ductal injury (Fig. 4A, B), following techniques described for complex biliary stone management [22].

## 6 Biliary drainage and completion

Following comprehensive stone clearance, a side-to-side choledochoduodenostomy was constructed with the first part of the duodenum using 3-0 Vicryl absorbable suture (Fig. 4D). This step was crucial for ensuring long-term biliary drainage and preventing recurrent obstruction, particularly given the history of recurrent pyogenic cholangitis, as supported by current evidence [19,23]. A cholecystectomy was then performed to complete the biliary intervention. Meticulous attention was paid to hemostasis, and a drain was positioned in the right subhepatic space for postoperative monitoring.

The total operative time was 280 min with minimal blood loss (<50 mL). No intraoperative complications were encountered, and complete stone clearance was achieved in the visualized ducts, though some small fragments remained in peripheral branches. A video demonstrating key aspects of the procedure is available as supplementary material.

## 7 Postoperative course

The patient's recovery was uneventful. Oral intake was resumed on postoperative day 2, and she was discharged on day 5. Laboratory values showed progressive improvement, with total bilirubin decreasing from 5.5 mg/dL to 1.2 mg/dL at discharge, and normalization of liver function tests.

**Fig. 5:**
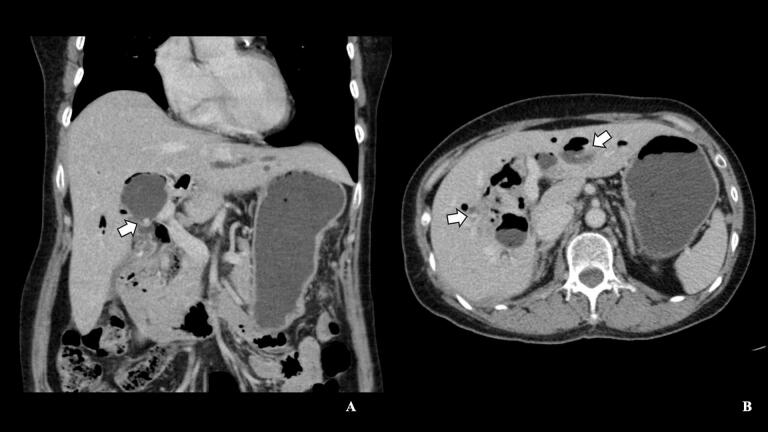
Postoperative CT scan at 6-month follow-up. (A) Coronal and (B) axial contrast-enhanced CT images at 6-month follow-up. Small retained stones (white arrows) are visible, but there is no significant biliary obstruction. The patent choledochoduodenostomy can be appreciated, indicating successful long-term biliary drainage.

At six-month follow-up, the patient remained asymptomatic with no recurrence of pain, fever, or jaundice. Follow-up computed tomography demonstrated patent biliary-enteric anastomosis with good drainage, though some small retained stones were noted in peripheral intrahepatic ducts (Fig. 5).

Given the significant risk of cholangiocarcinoma development in RPC patients, a structured surveillance protocol was implemented, including regular liver function tests and imaging studies [23,24]. The patent choledochoduodenostomy provides a pathway for potential spontaneous passage of retained fragments while maintaining adequate biliary drainage. The patient continues under systematic monitoring with no evidence of disease progression at current follow-up.

## 8 Discussion

This case demonstrates the successful application of laparoscopic choledochoscopy with thulium laser lithotripsy for managing complex hepatolithiasis in recurrent pyogenic cholangitis, highlighting both the technical advancements and ongoing challenges in treating this condition.

## 9 Evolution of minimally invasive management strategies

The therapeutic landscape for RPC and hepatolithiasis has evolved substantially to encompass multiple minimally invasive approaches. The development of advanced surgical techniques and technologies over the past three decades has enabled comprehensive management through various minimally invasive modalities, each with distinct advantages and limitations [7,24].

Endoscopic approaches represent a cornerstone in minimally invasive management. ERCP with stone extraction achieves success rates of 80–85 % for common bile duct stones, though its utility becomes limited when addressing peripheral intrahepatic stones [3,25]. Peroral cholangioscopy has emerged as a complementary technique, offering success rates of 57–71.4 % for intrahepatic stone management, particularly effective in cases involving smaller stones without significant biliary strictures. When combined with lithotripsy techniques, these endoscopic approaches can achieve stone clearance rates of up to 90 % in selected cases [19].

Percutaneous approaches, notably percutaneous transhepatic cholangioscopic lithotomy (PTCSL), demonstrate impressive initial stone clearance rates up to 85 % [12]. However, the high recurrence rates of 32–40 % highlight the importance of careful patient selection and the need for long-term follow-up strategies [8]. PTCSL presents a valuable option for high-risk surgical candidates or those with previous biliary surgery, particularly when combined with extracorporeal shockwave lithotripsy to improve stone clearance [26,27].

Laparoscopic management represents a significant advancement, with success rates reaching 94.1 % and notably low recurrence rates of 1.8 % [7]. Modern laparoscopic techniques enable both common bile duct exploration and anatomical liver resection, offering the advantages of minimally invasive surgery - reduced postoperative pain, shorter hospital stays, and faster recovery [28,29]. Recent meta-analyses have demonstrated non-inferiority of laparoscopic approaches compared to open surgery in terms of mortality, with superior outcomes in blood loss and length of stay [14].

The selection of specific minimally invasive techniques depends on multiple factors including stone location, presence of strictures, liver parenchymal status, and previous surgical history [24]. A recently developed difficulty scoring system for laparoscopic liver resection in hepatolithiasis helps guide appropriate patient selection and predict technical challenges [30]. Success ultimately relies on achieving three critical objectives: complete stone removal, establishment of adequate biliary drainage, and when indicated, resection of affected liver segments. This comprehensive approach aims to prevent stone recurrence and minimize the risk of disease progression [31,32].

## 10 Technical evolution in stone fragmentation

The progression of stone fragmentation technologies marks significant milestones in biliary stone management. Electrohydraulic lithotripsy (EHL), introduced in the early 1980s, represented the first major advance in minimally invasive stone fragmentation. Using a bipolar probe to generate shock waves through electrical discharge, EHL achieves success rates of 76–88 % and remains a cost-effective option when combined with cholangioscopy [22,33,34].

The advent of laser technology marked a significant improvement in precision and safety. Holmium:YAG systems, with over three decades of experience in urological applications, demonstrated success rates up to 97 % in biliary applications [35,36]. Key advantages include efficient stone fragmentation, minimal stone retropulsion, and reduced risk of ductal injury through the use of small-diameter fibers (200–1000 μm).

The emergence of thulium fiber laser technology represents the latest evolution, offering several distinct advantages [20,21]. Operating at a wavelength of 1940 nm, thulium laser demonstrates 4–5 times higher water absorption compared to Holmium systems, enabling more efficient stone fragmentation while minimizing collateral tissue effects [15,17]. Additionally, the technology produces remarkably fine fragments (<100 μm), potentially facilitating spontaneous passage and reducing the risk of retained stones [16].

## 11 Technical advantages of thulium laser technology

The thulium fiber laser system offers several technical advantages that make it particularly suitable for complex biliary stone management. The enhanced water absorption creates more efficient photothermal effects, leading to superior stone fragmentation while minimizing tissue penetration depth (0.2 mm compared to 0.4 mm for Ho:YAG) [15,17].

Our implementation of dual-setting parameters demonstrates the system's versatility:

‐ Dusting mode (0.2 J, 100 Hz, 20 W): Optimal for creating fine fragments, particularly effective for smaller stones and peripheral locations.
‐ Fragmentation mode (0.6–1.0 J, 30 Hz, 18 W): Designed for larger stones, providing controlled breakdown while maintaining safety margins.

Clinical experience suggests optimal outcomes with stones ranging from 1 to 2 cm in diameter, though the technology can effectively manage stones up to 4 cm with appropriate technique modification [15,21]. The most favorable results typically occur with stones in the 15–20 mm range, where the combination of dusting and fragmentation techniques can be most effectively employed [16,17].

## 12 Integration within comprehensive RPC management

The management of RPC requires a multifaceted approach extending beyond immediate stone clearance. Initial management of acute cholangitis remains paramount, followed by definitive stone clearance to prevent recurrent episodes, as outlined in current practice guidelines [23,37]. The creation of adequate biliary drainage through choledochoduodenostomy serves dual purposes: facilitating the passage of residual fragments and providing long-term protection against recurrent obstruction [19].

## 13 Long-term management considerations

The chronic nature of RPC necessitates comprehensive surveillance due to significant risks, particularly cholangiocarcinoma development with rates up to 21.2 % [6,24]. Key risk factors include age over 63 years, retained stones after treatment, and biliary strictures developing during follow-up. These risk factors guide surveillance protocols and intervention timing.

For asymptomatic residual stones without evidence of cholangitis, biliary strictures, or hepatic atrophy, active monitoring with regular imaging may be appropriate. Adjunctive medical therapy with ursodeoxycholic acid may be considered for small residual stones, though this does not replace the need for systematic imaging surveillance. The incidence of post-treatment intrahepatic stones ranges from 2.7 to 11 %, often resulting from impaired bile flow and biliary infection [24].

Our established surveillance protocol includes regular liver function tests, periodic imaging studies, and clinical assessment for early detection of complications. The patent biliary-enteric anastomosis facilitates spontaneous passage of residual fragments while maintaining adequate drainage, crucial for preventing stone reformation and recurrent cholangitis. This systematic approach to long-term management, combining regular monitoring with appropriate intervention when needed, is essential for optimizing outcomes in this challenging condition.

## 14 Implementation and economic considerations

The adoption of thulium laser technology requires careful consideration of several practical factors. While the initial investment in equipment and specialized training represents a significant commitment, potential benefits include reduced operative time, decreased complication rates, and improved stone clearance efficiency [17]. The learning curve associated with this technology necessitates structured training programs and careful case selection during initial implementation, as supported by current evidence [15].

## 15 Future directions

Several critical areas warrant further investigation to optimize this therapeutic approach. The refinement of laser parameters specifically for biliary applications remains an important research focus, as current settings are largely adapted from urological experience [16,17]. Long-term comparative studies between different lithotripsy modalities would provide valuable data on relative efficacy and cost-effectiveness [15]. The development of standardized protocols for staged management of complex cases could help address the challenge of complete stone clearance, particularly in patients with difficult anatomical configurations or extensive stone burden [19].

## 16 Conclusion

The successful application of laparoscopic choledochoscopy with thulium laser lithotripsy demonstrates a promising minimally invasive option for managing complex hepatolithiasis in recurrent pyogenic cholangitis. While this approach offers advantages in stone fragmentation efficiency and reduced surgical trauma, it represents one component within a comprehensive RPC management strategy rather than a definitive cure.

The presence of retained stones in our case highlights both the technical challenges of managing complex hepatolithiasis and the importance of establishing adequate long-term biliary drainage. The creation of a patent biliary-enteric anastomosis provides a pathway for spontaneous passage of residual fragments while maintaining long-term drainage, particularly important given the significant risk of cholangiocarcinoma development in this patient population.

Our experience suggests promise for thulium laser technology in complex biliary stone management, though several areas require further investigation through controlled studies, including optimization of laser parameters for biliary applications and evaluation of long-term outcomes. Success ultimately depends on appropriate patient selection, technical expertise, and implementation of comprehensive follow-up strategies.

The following is the supplementary data related to this article.

Video 1 Laparoscopic thulium laser-assisted management of intrahepatic stones with choledochoduodenostomy. Video 1

## Author contribution

Hariruk Yodying: Conceptualization, data collection, writing - original draft, review and editing.

## Consent

Written informed consent was obtained from the patient for publication of this case report and accompanying images. A copy of the written consent is available for review by the Editor-in-Chief of this journal on request.

## Ethical approval

Ethical approval was not required for this single case report as per the institutional guidelines of Srinakharinwirot University. However, we have adhered to our institutional policies for case reports, including obtaining written informed consent from the patient for publication of this case report and accompanying images. We have completed the institutional ethics form for case reports (Form AF26-03-03.0) as required by our institution. Patient privacy and confidentiality have been maintained throughout the report.

## Guarantor

Hariruk Yodying.

## Funding

This research did not receive any specific grant from funding agencies in the public, commercial, or not-for-profit sectors.

## Conflict of interest statement

The authors declare that they have no known competing financial interests or personal relationships that could have appeared to influence the work reported in this paper.
